# Transcriptomic Analysis of Newborn Hanwoo Calves: Effects of Maternal Overnutrition during Mid- to Late Pregnancy on Subcutaneous Adipose Tissue and Liver

**DOI:** 10.3390/genes15060704

**Published:** 2024-05-28

**Authors:** Borhan Shokrollahi, Hyun-Jeong Lee, Youl Chang Baek, Shil Jin, Gi-Suk Jang, Sung Jin Moon, Kyung-Hwan Um, Sun Sik Jang, Myung Sun Park

**Affiliations:** 1 Hanwoo Research Institute, National Institute of Animal Science, Pyeongchang 25340, Republic of Korea; borhansh@gmail.com (B.S.); chang4747@korea.kr (Y.C.B.); jins21@korea.kr (S.J.); shinnanda16@naver.com (G.-S.J.); moonsj27@korea.kr (S.J.M.); umkh9969@korea.kr (K.-H.U.); 2 Animal Nutrition and Physiology Division, National Institute of Animal Science, Rural Development Administration, Wanju 55365, Republic of Korea; hyunj68@korea.kr

**Keywords:** maternal overnutrition, Hanwoo newborn calves, subcutaneous adipose tissue, liver, RNA sequencing

## Abstract

This study investigated the transcriptomic responses of subcutaneous adipose tissue (SAT) and liver in newborn Hanwoo calves subjected to maternal overnutrition during mid- to late gestation. Eight Hanwoo cows were randomly assigned to control and treatment groups. The treatment group received a diet of 4.5 kg of concentrate and 6.5 kg of rice straw daily, resulting in intake levels of 8.42 kg DMI, 5.69 kg TDN, and 0.93 kg CP—higher than the control group (6.07 kg DMI, 4.07 kg TDN, and 0.65 kg CP), with respective NEm values of 9.56 Mcal and 6.68 Mcal. Following birth, newly born calves were euthanized humanely as per ethical guidelines, and SAT and liver samples from newborn calves were collected for RNA extraction and analysis. RNA sequencing identified 192 genes that were differentially expressed in the SAT (17 downregulated and 175 upregulated); notably, *HSPA6* emerged as the most significantly upregulated gene in the SAT and as the singular upregulated gene in the liver (adj-*p* value < 0.05). Additionally, differential gene expression analysis highlighted extensive changes across genes associated with adipogenesis, fibrogenesis, and stress response. The functional enrichment pathway and protein–protein interaction (PPI) unraveled the intricate networks and biological processes impacted by overnutrition, including extracellular matrix organization, cell surface receptor signaling, and the PI3K-Akt signaling pathway. These findings underscore maternal overnutrition’s substantial influence on developmental pathways, suggesting profound cellular modifications with potential lasting effects on health and productivity. Despite the robust insights that are provided, the study’s limitations (sample size) underscore the necessity for further research.

## 1. Introduction

During pregnancy, complex physiological and metabolic changes occur to support both the dam’s metabolism and the growing fetus [1]. Maternal nutrition imbalances affect energy distribution among metabolically active tissues such as the liver and SAT in the offspring [2,3]. Such imbalances during this period can induce responsive and adaptive changes in fetal development [4], potentially influencing organ structures, functions, and metabolism later in life [5,6], and could be epigenetically transmitted across generations [7]. Therefore, ensuring adequate maternal nutrition during pregnancy is crucial for shaping fetal and long-term developmental programming [8], influencing the offspring’s health and productivity [6,9,10].

Studies have highlighted that maternal overnutrition impacts gene expression differently across various tissues. Specifically, modifications in nutrition around the time of birth, particularly in protein and energy levels, are linked to decreased gene expression concerning muscle growth and differentiation in the *Longissimus dorsi* muscle of calves [11]. Another study showed that maternal fatty acid supplementation influenced specific gene expressions, such as *MYH7* and C/*EBP*β in muscle, and *ZFP423* in adipose tissue of beef calves [12]. Moreover, a study revealed that maternal protein supplementation during mid-gestation led to the differential expression of 310 genes in 260-day-old calves, notably enhancing genes involved in insulin signaling and apoptosis [13].

In addition, maternal nutrition also significantly affects adipose tissue. The literature has demonstrated that altering maternal nutrition, including both over- and undernutrition during gestation, influences adipose tissue development and the expression of adipogenesis marker genes in fetuses [14,15], underscoring the significant role of maternal nutrition during gestation in fetal adipose tissue development. The period of mid- to late gestation is recognized as pivotal to adipose tissue development [8]. Underwood et al. [16] proposed that the nutritional status during mid-gestation could influence the deposition of subcutaneous adipose tissue in steers destined for slaughter weights. Zhang et al. [17] found that providing cows with different levels of nutrition during gestation affected SAT accumulation in Wagyu cow fetuses at 260 days of fetal age, with those receiving overnutrition having larger fat depots compared to those receiving restricted nutrition. In terms of SAT, fetuses from low-nutrition diets showed an increased expression of leptin and *PPARG* mRNA. Furthermore, a study observed significant differences in gene expression and adipocyte size between fetuses exposed to maternal overnutrition and those from low-nutrition groups in Wagyu cows, particularly in the SAT, and fetuses from the overnutrition group exhibited a larger adipocyte diameter [18]. Likewise, in another study, it was reported that maternal overnutrition in Angus-crossbred heifers from day 85 to day 180 of gestation had a significant effect on fetal depots, including the SAT and the expression of preadipocyte factor-1 (*PREF*-1) [19]. Long et al. [20] observed that calves exposed to maternal diets providing only 55% of normal nutritional levels during early gestation exhibited reduced expression of the *AP2*, *CD36*, and *GLUT4* genes in perirenal adipose tissue. Another study in sheep showed that maternal overnutrition of 150% increased SAT mass and leptin mRNA levels, although other genes, like *PPAR*-γ, adiponectin, *LPL*, and *G3PDH,* did not differ significantly in lambs from the overfed dams [21].

Similarly, the fetal liver—an essential metabolic organ—also shows marked changes in response to maternal diet. Various biological mechanisms, including gene expression, regulate the development of the fetal liver [22]. Nutritional signals activate pathways that regulate gene activity, thereby influencing cell growth and differentiation [4,22]. An increasing body of research utilizing ewes and cows has underscored the impact of maternal nutrition during mid- to late gestation on fetal liver development and function [10,23,24,25]. Prezotto et al. [26] observed an increase in liver weight in fetuses from nutrient-restricted and realimented beef cows compared to the control group. On the other hand, Diniz et al. [27] found that administering vitamin and mineral supplements from at least 71 days before breeding until day 83 of gestation influences fetal hepatic function by changing the expression of genes associated with energy and lipid metabolism. Another study on maternal undernutrition in fetal calves demonstrated that calves whose mothers were fed 60% of their nutritional requirements exhibited significant hypomethylation in liver genes compared to those fed 120% [28]. These studies underline the important influence of maternal nutrition on fetal liver gene expression, emphasizing how dietary variations during pregnancy can significantly shape the developmental and metabolic pathways in offspring.

To date, studies exploring the impact of maternal overnutrition on the liver and SAT transcriptomes in newborn beef cattle remain limited. In this study, we hypothesize that maternal overnutrition during mid- to late pregnancy leads to changes in the transcriptomics of the SAT and liver in newborn Hanwoo calves, affecting their metabolic pathways.

## 2. Materials and Methods

### 2.1. Ethical Approval

The research protocol was examined and sanctioned by the Institutional Animal Care and Use Committee at the National Institute of Animal Science (NIAS), documented under the reference NIAS2021-0526 (20 May 2021). The care and experimental engagement with Hanwoo cows and their offspring were rigorously in line with the Hanwoo Research Institute’s (NIAS) established ethical guidelines. All animal management and experimental activities were performed in strict adherence to these standards, guaranteeing the well-being and ethical treatment of the animals involved. Measures were proactively taken to reduce any potential distress or discomfort to the animals throughout the research duration, with immediate corrective actions implemented for any deviations from the approved protocols.

### 2.2. Animals, Dietary Regimens, and Management Practices

This investigation was conducted at the Hanwoo Research Institute, part of the National Institute of Animal Science, spanning a period of 10 months from June 2021 to April 2022. The study involved eight Hanwoo cows, selected randomly from the pregnant research cows. Before artificial insemination, all cows were managed uniformly and received a baseline diet to standardize their nutritional intake according to the guidelines of Korean feeding standards for Hanwoo [29]. Following insemination, the cows were randomly assigned into two dietary groups of four. The age, weight, and body condition score (BCS) showed no significant differences between the groups at the start and middle of the pregnancy; however, an increase in BCS was observed in the overfed cows during the late stage of pregnancy [30]. The details regarding the number of inseminations required per pregnancy averaged 1.5, and pregnancy was initially confirmed 90 days after artificial insemination using a pregnancy diagnosis kit, followed by a manual examination to verify pregnancy.

Both dietary groups were initially fed identical rations of rice straw and concentrate. The control group continued to receive a steady diet comprising 3 kg of concentrate and 5 kg of rice straw daily throughout their gestation. This aligns with the Korean Feeding Standards for breeding Hanwoo, which specifies the maintenance energy requirements for a 400 kg cow as a Dry Matter Intake (DMI) of 4.71 kg, Total Digestible Nutrients (TDN) intake of 2.12 kg, and Crude Protein (CP) intake of 0.47 kg. In our experiment, the control group’s actual intake was set at a DMI of 6.07 kg, 4.07 kg TDN, and 0.65 kg CP, representing a typical feeding level for pregnant cows according to these standards [29]. In contrast, the treatment group, simulating overnutrition, received an elevated diet during mid-to-late gestation of 4.5 kg concentrate and 6.5 kg rice straw daily, representing a 150% increase in feed. The treatment group was intentionally overfed, receiving a DMI of 8.42 kg, 5.69 kg TGN, and 0.93 kg CP, resulting in a net energy for maintenance (NEm) of 9.56 Mcal, compared to 6.68 Mcal for the control group. This nutritional strategy was employed to assess the impact of overfeeding on fetal development, focusing on metabolic and physiological adaptations in response to an increased nutritional intake. The dietary compositions were analyzed according to the AOAC (2004) guidelines. Details about the composition diets can be found in Table 1.

The management of experimental cows strictly adhered to the ethical and welfare standards set by the Hanwoo Research Institute. Nutritional feed was provided twice daily, at 8 a.m. and 4 p.m., with unrestricted access to water and mineral supplements available around the clock.

### 2.3. Collection and Preservation of SAT and Liver Samples from Newborn Calves

Following birth, newborn male calves (30.9 kg) were immediately separated from their mothers, ensuring there was no colostrum intake, in line with the study’s experimental design. The calves were then humanely euthanized using a protocol approved by the Institutional Animal Care and Use Committee, involving the administration of pentobarbital intravenously at a dose of 100 mg/kg body weight [31] to ensure the rapid and humane cessation of life functions. This method, followed by exsanguination, was carefully chosen to align with the international standards for ethical animal treatment, ensuring minimal animal distress and the preservation of tissue integrity that was essential for our molecular analyses. SAT and liver samples were then harvested during necropsy. SAT was specifically collected from the rump area, and each sample consistently weighed approximately 20 g. Liver samples were carefully extracted from the right lateral lobe, a common choice for standardized sampling due to its accessibility and size, and each sample ranged in weight from 20 to 30 g. The samples were labeled according to the mother’s ear tag number. These tissues were promptly transported to a laboratory setting, where they were frozen at a temperature of −80 °C to halt biochemical processes, thereby preserving the integrity of the samples for subsequent molecular analyses.

### 2.4. RNA Isolation, Quality Assurance, and Sequencing Protocol

Total RNA was extracted from samples of newborn calf SAT and liver using the TRIzol reagent protocol, emphasizing the prevention of RNA degradation and contamination. The integrity of the RNA was meticulously evaluated using an Agilent TapeStation with RNA screentape (Agilent, #5067-5576, Agilent Technologies, Santa Clara, CA, USA), ensuring that only RNA samples with an Integrity Number (RIN) of 7.0 or higher were advanced to the next stage. For library construction, 0.5 μg of high-quality total RNA from each sample was processed using the Illumina TruSeq Stranded Total RNA Library Prep Globin Kit (Illumina, Inc., San Diego, CA, USA). This process began with the depletion of ribosomal RNA (rRNA) from the total RNA samples, employing the Ribo-Zero rRNA Removal Kit (Illumina, Inc., San Diego, CA, USA). Subsequent steps involved fragmenting the mRNA into smaller pieces, synthesizing first-strand cDNA using SuperScript II reverse transcriptase (Invitrogen, #18064014, Invitrogen, Carlsbad, CA, USA) and random primers, and generating second-strand cDNA. The cDNA fragments underwent end repair, A-tailing, and adapter ligation, and were enriched through PCR to finalize the cDNA library. The prepared libraries were quantified using KAPA Library Quantification kits (KAPA BIOSYSTEMS, #KK4854, KAPA BIOSYSTEMS, Wilmington, MA, USA) and assessed for quality with the TapeStation D1000 ScreenTape (Agilent Technologies, #5067-5582). Indexed libraries were sequenced on an Illumina NovaSeq platform, executing paired-end (2 × 100 bp) sequencing facilitated by TNT Research Incorporated (Seoul, Republic of Korea). 

### 2.5. Transcriptomic Data Preprocessing and Analysis

Upon sequencing, raw data underwent an initial quality control to eliminate adapter sequences and low-quality reads. This was accomplished using FastQC v0.11.7 [32] for preliminary assessment and Trimmomatic for trimming tasks, where adapter sequences were removed and reads were trimmed based on quality thresholds (base quality < 3, sliding window size 4, average quality > 15, minimum length of 36 bp). The cleaned reads were then aligned with the reference bovine genome (ARS-UCD1.3), utilizing Hierarchical Indexing for Spliced Alignment of Transcripts 2 (HISAT2) version 2.1.0 [33]. Following alignment, StringTie version 2.1.3b facilitated transcript assembly and provided quantification metrics such as Fragments Per Kilobase of transcript per Million mapped reads (FPKM) and Transcripts Per Million (TPM), which are critical to the differential expression analysis. This comprehensive workflow ensured the accurate identification of differentially expressed genes (DEGs) based on rigorous statistical analysis, setting the foundation for deep biological insights into the transcriptomic responses to maternal overnutrition in newborn calves.

### 2.6. Analysis of Differential Gene Expression, Gene Ontology (GO), and Pathway Enrichment

DESeq2 [34] was utilized for the statistical analysis of gene expression differences, employing raw count data and prioritizing findings with an adjusted *p*-value below 0.05. Data visualization was achieved using R packages, such as ggplot2 for creating volcano plots and pheatmap for generating heatmaps. These visualizations helped to interpret expression patterns and organize sample clustering. A functional enrichment analysis was conducted using g:Profiler for GO analysis and to explore the roles of DEGs in biological processes, cellular components, molecular functions, and the Kyoto Encyclopedia of Genes and Genomes (KEGG) pathways [35].

### 2.7. Protein–Protein Interaction (PPI) Network Analysis

To dissect the intricate network of protein interactions stemming from the DEGs, we employed the STRING database (version 12.0), which amalgamates experimental, computational, and textual evidence, to map protein interactions. Inputting the DEGs from the SAT and liver, we explored their potential physical and functional connections, setting a confidence threshold at 0.7 to ensure the analysis’s relevance and reliability [36].

### 2.8. Data Analysis

A differential expression analysis was conducted using DESeq2 within the R statistical environment, focusing on identifying genes with significant expression changes between control and treatment groups. The criteria for statistical significance were based on the adjusted *p*-values, with a threshold set at <0.05, following the Benjamini–Hochberg procedure to control the false discovery rate (FDR) due to the multiple tests. This analysis framework ensured that the reported DEGs were identified with high confidence, minimizing the risk of false positives. Functional enrichment and pathway analyses of these DEGs were performed using g:Profiler to elucidate their biological implications and the pathways potentially affected by maternal overnutrition. This comprehensive approach provided a robust statistical foundation for our transcriptomic analysis, enabling the reliable interpretation of the effects of maternal nutrition on newborn development.

## 3. Results

### 3.1. Analysis of RNA Sequencing Data

Sixteen samples from the SAT and liver of newborn Hanwoo calves were thoroughly evaluated to explore the impacts of excessive maternal nutrition during mid- to late gestation. The RNA RIN for these samples was between 7 and 8, underscoring the extracted RNA’s high quality. RNA sequencing generated a total of 641.9 million paired-end reads, averaging about 65 million reads for the SAT and 54.5 million reads for liver tissue. Impressively, 99% of the total reads were precisely aligned with the bovine reference genome, showcasing the high efficiency of the genomic mapping process in this study. However, due to RNA sequencing technicalities, the analysis was limited to a reduced subset of the initially collected samples. Specifically, the SAT analysis was conducted on one treated sample and three control samples, due to the limited availability of high-quality RNA. For liver tissue analysis, three treated samples and four control samples were used, reflecting the higher availability of samples meeting our stringent quality criteria.

### 3.2. Analyzing Differential Gene Expression

**Figure 1:**
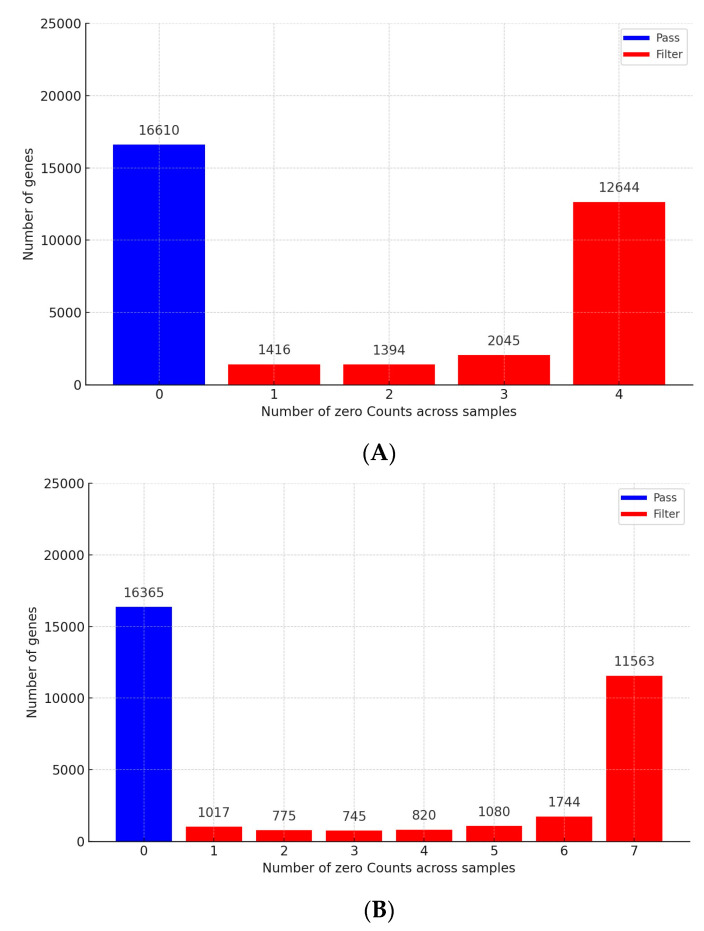
Gene filtering based on zero counts in the SAT and liver: Panel (**A**) depicts the process of gene exclusion due to zero counts in the SAT, while Panel (**B**) illustrates the analogous procedure for liver.

This investigation undertook an in-depth analysis of the gene expression variability among 32,975 genes within the SAT and liver tissues of newborn calves to uncover the impact of maternal overfeeding. A substantial number of genes, amounting to 17,499 in SAT and 17,744 in liver tissue, were found to have one or more instances of zero counts and were subsequently omitted from the analysis. The process of gene removal for each tissue type is graphically depicted in Figure 1A,B, while a detailed list of all evaluated genes is cataloged in Table S1.

**Figure 2:**
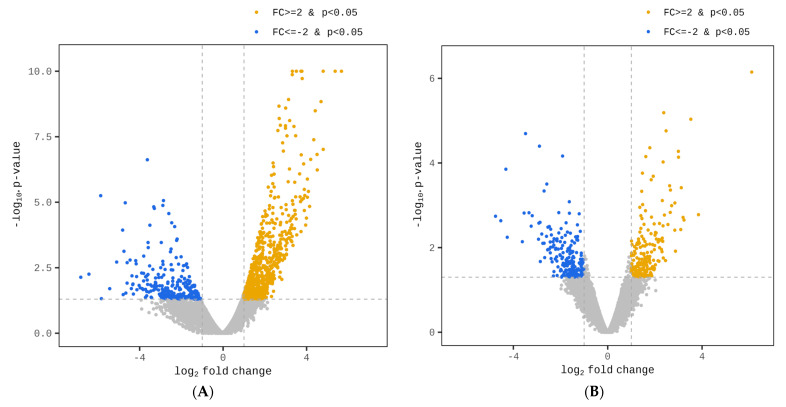
Volcano plots depicting the influence of maternal overnutrition during mid- to late gestation on the gene expression in subcutaneous adipose tissue (SAT) (**A**) and liver (**B**) of newborn Hanwoo calves. The Y-axis illustrates the negative logarithm of *p*-values (−log10 *p*-values), and the X-axis represents the fold change (FC; log_2_ fold change). Blue dots to the left signify downregulated, differentially expressed genes (DEGs), while orange dots to the right mark upregulated DEGs. DEGs significantly affected by maternal overnutrition (*p*-value < 0.05) are emphasized.

A subsequent analysis of the SAT identified significant expression differences in 983 genes, marked by a fc > |2| and a *p* < 0.05. Within this group, 731 genes exhibited upregulation, while 252 genes had a downregulated expression. In the liver tissue, 408 genes were found to conform to the same criteria of a fold change and *p*-value. Amongst these, 197 genes were more highly expressed and 211 showed a reduced expression. Figure 2 displays volcano plots illustrating the impact of overnutrition on gene expression within the SAT and liver of newborn Hanwoo calves.

**Figure 3:**
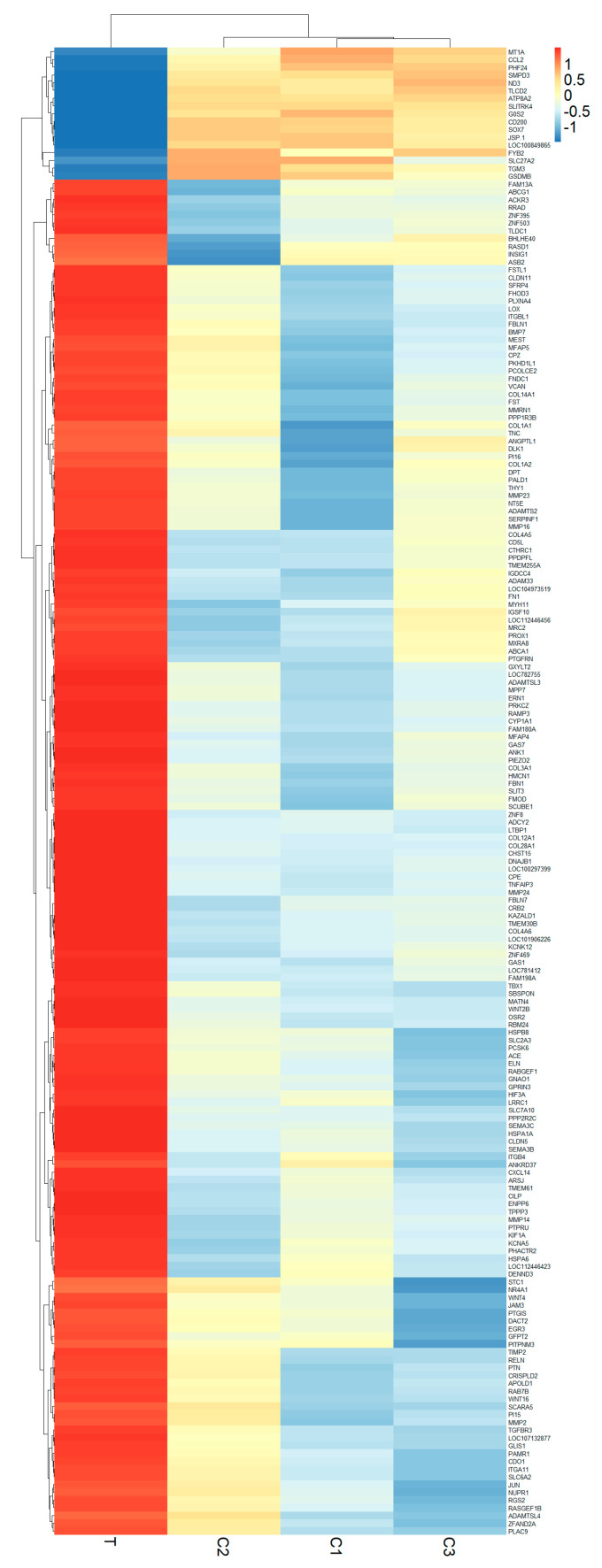
Heatmaps illustrating DEGs in the SAT of newborn Hanwoo calves. Within the heatmap, columns correspond to individual samples, where sample T was from the treatment group and samples C1, C2, and C3 were from the control group. A dendrogram is provided to display the overall expression trends visually. The spectrum of colors from red to blue signifies the extent of gene upregulation and downregulation, respectively.

In the SAT, a subset of 192 genes exhibited notable expression differences (with an adjusted *p*-value < 0.05), underscoring the nuanced regulatory changes induced by maternal overnutrition. Of these DEGs, 175 were upregulated, while 17 were downregulated (Table S1). This highlights the significant transcriptional shifts towards increased gene activity within the SAT in response to overnutrition. Remarkably, the liver tissue displayed a singular significantly upregulated DEG, identified as *HSPA6*, showcasing a fold change of 68.7, which also emerged as the highest upregulated DEG in the SAT, with a fold change of 50.9. This parallel elevation underscores the systemic impact of maternal diet on crucial metabolic tissues. The differential gene expression patterns of SAT DEGs (adjusted *p*-value < 0.05) are illustrated through heatmaps in Figure 3. Notably, the top 10 upregulated DEGs in the SAT, including *HSPA6*, *CYP1A1*, *LOC781412*, *LOC112446423*, *ITGBL1*, *ENPP6*, *FAM198A*, *PPDPFL*, *PRKCZ*, and *PI15*, reflect a diverse array of biological functions and pathways potentially impacted by maternal overnutrition.

### 3.3. GO Enrichment Analysis

An in-depth GO analysis was conducted to explore the biological roles of DEGs in the SAT and liver tissues of newborn Hanwoo calves. For the SAT, we performed a GO analysis of 192 genes that showed significant expression differences with an adjusted *p*-value < 0.05. However, in the liver, only one DEG showed significant expression at the same significance level; a broader analysis approach was adopted, accepting DEGs with a *p*-value < 0.01 to explore their biological significance. In total, these analyses created 3883 and 2454 categories for the SAT and liver, respectively.

**Figure 4:**
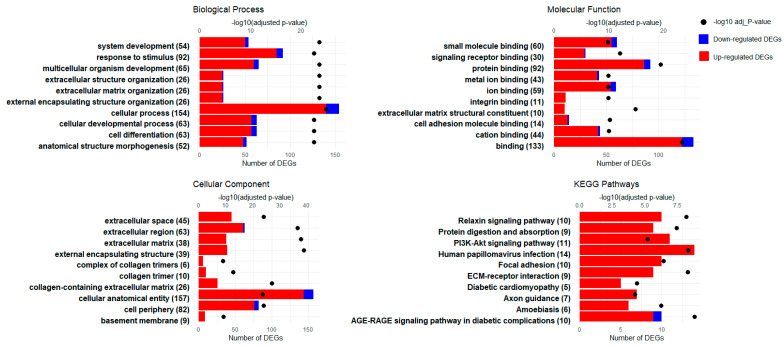
GO enrichment and KEGG pathway analysis for the SAT: This figure showcases the outcomes of both Gene Ontology (GO) enrichment and KEGG pathway analyses, focusing on the top 10 identified terms across biological processes, molecular functions, and cellular components, as well as the key KEGG pathways affected in the SAT as a result of maternal overnutrition. The biological processes depict general categories where gene expression is modified, the molecular functions show changes in protein activity or binding capabilities, and the cellular components indicate the locations within the cell where these changes occur. The KEGG pathways offer insights into the complex metabolic and signaling pathways that are affected. The Y-axis indicates the number of differentially expressed genes (DEGs), with blue and red bars representing downregulated and upregulated DEGs for each identified term, respectively, providing an indication of the directionality of the expression changes. The X-axis shows the number of DEGs associated with each of the top 10 GO terms and KEGG pathways. On the opposing Y-axis, the −log10 adjusted *p*-values for each term are illustrated by dots, providing a measure of significance for the enrichment of each term.

The GO enrichment analyses of the biological processes, molecular functions, and cellular components in the SAT of newborn Hanwoo calves present a comprehensive picture of the tissue’s adaptation to maternal overnutrition. Central to these changes is the remodeling of the extracellular matrix (ECM), underscored by the upregulation of structural genes like *FBLN1*, *LOX*, and various collagens, pointing to the extensive restructuring of the cellular environment (GO:0030312, GO:0031012). This process is fundamental to not only the tissue architecture but also cellular signaling, as evidenced by the enrichment of genes in “protein binding” (GO:0005515) and “signaling receptor binding” (GO:0005102), reflecting a recalibration of the cellular communication and metabolic responsiveness pathways. Such modifications are instrumental for orchestrating developmental processes, including multicellular organism development (GO:0007275), system development (GO:0048731), and cell differentiation (GO:0030154), which are critical for growth and function. The emphasis on cell–environment interfaces, including the “cell periphery” (GO:0071944) and “basement membrane” (GO:0005604), further highlights the dynamic nature of cellular interactions and the importance of integrin binding (GO:0005178) in response to nutritional cues (Figure 4 and Table S2). Collectively, the SAT’s response to overnutrition suggests an adaptive strategy affecting not only the structural matrix but also the molecular pathways essential for tissue development, differentiation, and metabolic regulation, highlighting the far-reaching impact of maternal diet on calf development.

**Figure 5:**
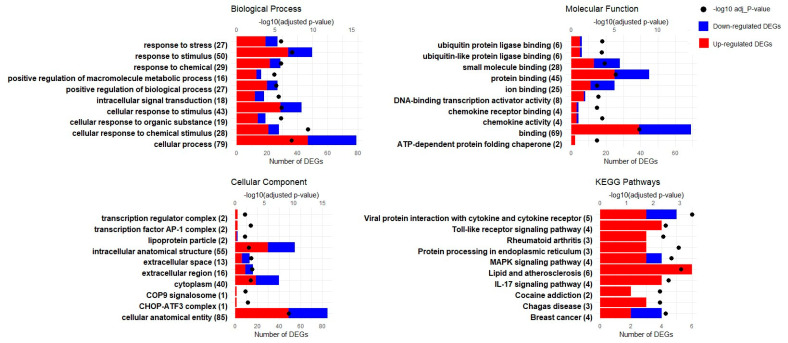
GO enrichment and KEGG pathway analysis for liver: This figure showcases the outcomes of both Gene Ontology (GO) enrichment and KEGG pathway analyses, focusing on the top 10 identified terms across biological processes, molecular functions, and cellular components, as well as key KEGG pathways. The Y-axis indicates the number of differentially expressed genes (DEGs), with blue and red bars representing downregulated and upregulated DEGs for each identified term, respectively. The X-axis details the top 10 GO terms and KEGG pathways. On the opposing Y-axis, the −log10 adjusted *p*-values for each term are illustrated by dots, providing a measure of significance for the enrichment of each term.

The GO enrichment analysis of the liver from Hanwoo calves exposed to maternal overnutrition reveals critical adaptations within several key domains: biological processes such as “cellular response to chemical stimulus” (GO:0070887) and “regulation of transcription in response to stress” (GO:0043618) indicate a heightened state of metabolic alertness and stress management. Molecular function changes, particularly in transporter activity (GO:0015347), suggest modified metabolic clearance processes. Cellular component shifts in the “transcription factor AP-1 complex” (GO:0035976) point to a reprogramming of the liver’s transcriptional response to stress (Figure 5 and Table S3). These responses, encompassing a variety of cellular mechanisms, highlight the liver’s complex role in adapting to increased nutritional intake and maintaining metabolic balance.

### 3.4. KEGG Pathway Enrichment Analysis

A KEGG pathway analysis was performed to evaluate the pathways involved in the SAT and liver tissues of newborn Hanwoo calves due to maternal overnutrition. Similar criteria were applied for both tissues to those used in the GO analysis. As a result, this analysis identified 191 pathways for SAT and 113 pathways for liver.

The KEGG pathway analysis of the SAT from Hanwoo calves responding to maternal overnutrition emphasizes alterations in key pathways that are fundamental to growth and metabolism. These include the “ECM–receptor interaction” (KEGG:04512), with crucial genes such as *COL1A1* and *COL1A2*, underscoring changes in tissue connectivity and integrity. The “PI3K-Akt signaling pathway” (KEGG:04151), featuring genes like *ITGA11* and *NR4A1*, suggests modifications in growth signals and metabolic processes. “Focal adhesion” (KEGG:04510), which involves *ITGB4* and *TNC*, points to shifts in cellular interactions and signaling. The “Relaxin signaling pathway” (KEGG:04926) with *PRKCZ* and *MMP2* indicates a role in inflammation and extracellular matrix remodeling. Lastly, the “AGE-RAGE signaling pathway in diabetic complications” (KEGG:04933) is also enriched, implicating the collagen genes in advanced glycation end-product-related cellular responses, which are relevant for metabolic dysregulation in the SAT (Figure 4). These pathways collectively reflect the tissue’s response to an overnutritive environment, impacting the developmental and metabolic pathways.

The KEGG pathway enrichment in the liver of Hanwoo calves exposed to maternal overnutrition emphasizes the critical stress response and signaling pathways. The “protein processing in endoplasmic reticulum” (KEGG:04141) and “MAPK signaling pathway” (KEGG:04010) are notably affected, suggesting an enhanced cellular response to protein management and inflammatory signaling, with key genes like *HSPA1A* and *JUN* being central to these processes. Pathways like “Lipid and atherosclerosis” (KEGG:05417) and “IL-17 signaling” (KEGG:04657) underline potential shifts in lipid metabolism and immune responses, indicative of the liver’s complex reaction to nutritional excess (Figure 5).

### 3.5. Protein–Protein Interaction Analysis of SAT and Liver Tissues

**Figure 6:**
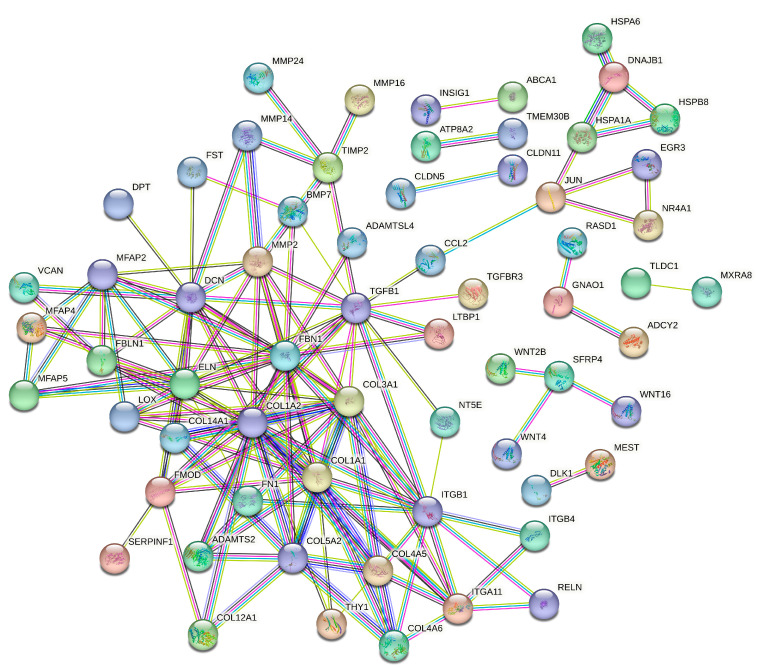
Protein–Protein Interaction (PPI) Network for the SAT: This network illustrates the interactions between proteins encoded by DEGs as a response to maternal overnutrition. Nodes symbolize the individual proteins, while the connecting lines (edges) indicate the protein interactions.

The PPI network analysis for the SAT and liver were meticulously conducted to decipher the complex web of protein interactions that could illuminate the molecular mechanisms underlying the response to maternal overnutrition. For the SAT, using 192 significant DEGs, the results highlight a network including 184 proteins (nodes) connected by 142 edges. The network’s structure is characterized by an average node degree of 1.54, and a clustering coefficient of 0.252, suggesting a dense yet moderately clustered interaction pattern, which may indicate functional groupings or pathways that are crucial for adipose tissue response to overnutrition. Prominent central nodes within the network included key structural proteins such as *COL1A1*, *COL1A2*, *COL3A1*, and *COL6A2*, which emerged as significant hubs, indicative of their roles in extracellular matrix integrity. These proteins, along with *ADAMTS2*, which plays a key role in procollagen processing, and chemokine *CCL2,* which links structural components and signaling pathways, underline a complex interplay that could be pivotal in metabolic regulation and inflammatory responses in the SAT of neonatal calves. This mapping of interactions provides foundational insights into the biological processes and potential disease mechanisms influenced by maternal nutrition (Figure 6).

**Figure 7:**
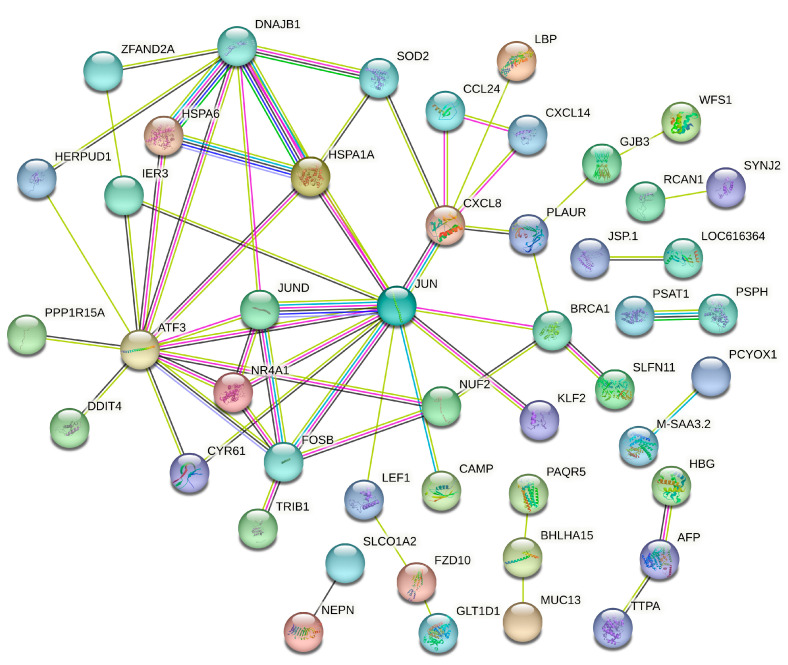
Protein–Protein Interaction (PPI) Network for the liver: This network illustrates the interactions between proteins encoded by DEGs as a response to maternal overnutrition. Nodes symbolize the individual proteins, while the connecting lines (edges) indicate the protein interactions.

For the liver, the PPI analysis incorporated 129 significant DEGs (*p* < 0.01) to assemble a network of 103 proteins (nodes) connected by 61 edges, revealing a less connected yet functionally significant interaction pattern. This intricate network displays an average node degree of 2.04, meaning that each protein typically interacts with two other proteins. The network’s average local clustering coefficient is 0.341. Proteins like ATF3, FOS, and JUN, with high degrees of connectivity, are known to play significant roles beyond the general stress response, such as in endothelial vascular disease and cellular adaptation to stress, contradicting the notion of their being involved only in generic stress responses. Their high connectivity in the liver network suggests a nuanced role in liver adaptation to overnutrition, potentially influencing pathways related to inflammation and stress protection, as highlighted by proteins such as *CXCL8* and the HSPs *HSPA6* and *HSPA1A* (Figure 7). The depiction of these networks in Figure 6 and Figure 7 aims to provide a visual interpretation of the complex interactions, with an enhanced focus on readability and the inclusion of key proteins to aid in the understanding of their specific roles and interconnections.

## 4. Discussion

Maternal nutrition significantly shapes fetal development and meat quality in cattle, with studies indicating that better nutrition improves fetal body. An enhanced SAT due to an improved maternal diet contributes to better meat quality by positively influencing the activity of fibroblasts and fibro-adipogenic progenitors, which play a critical role in marbling and the overall tenderness of the meat [18,37]. Additionally, the fetal liver holds a pivotal position in sustaining metabolic balance [2,22]. This study explored how maternal overfeeding during mid- to late gestation affects gene expression in the SAT and liver of newborn Hanwoo calves. Overnutrition during the pregnancy period can lead to metabolic stress in the developing fetus [38], leading to significant biological responses. Our findings demonstrate that the *HSPA6* gene is the most upregulated in both the SAT and liver, with *HSPA1A* also showing significant upregulation in the SAT. This pronounced gene expression highlights their crucial role in mitigating the metabolic stress induced by maternal overfeeding.

Heat shock proteins (HSPs), particularly the *HSP70* family members including *HSPA6*, act as cellular guardians under stress conditions. They play a vital role in maintaining proteostasis by assisting in the refolding of denatured proteins and preventing protein aggregation, thereby preserving cell viability and function [39,40]. Moreover, the universal stress marker role of *HSP70* highlights its sensitivity to various stress conditions, including metabolic stress induced by overnutrition [41]. The elevated expression of *HSPA6* in conditions of thermal stress, as reported in goats [42,43], underscores our findings in bovine fetal tissues due to maternal overfeeding, suggesting a conserved stress-response mechanism across species. This implies that *HSPA6*’s upregulation may serve as a critical adaptive response to metabolic imbalances caused by maternal overnutrition, aiming to restore cellular homeostasis and energy balance.

Furthermore, the review by Kuppuswami and Senthilkumar [40] elucidates the complex relationship between HSPs and mitochondrial integrity. Given the centrality of the mitochondria in energy metabolism and their vulnerability to nutri-stress, the protective role of HSPs in maintaining mitochondrial function becomes paramount. This suggests that *HSPA6* may also play a crucial role in protecting mitochondrial integrity in fetal tissues exposed to maternal overnutrition, thereby mitigating the risk of metabolic dysfunction. Typically, *HSPA6* expression remains low in most tissues under non-stressful conditions, with notable exceptions in certain blood cells, highlighting its specific role in stress response [44]. This suggests that the observed upregulation of *HSPA6* in our study may signal a metabolic stress response in fetal tissues due to maternal overnutrition. In light of the evidence pointing towards a genetic basis for stress adaptability, as indicated by the differential expression of *HSPA6* in response to heat stress in cattle breeds [45], our findings may also hint at a genetic predisposition towards metabolic stress resilience in neonatal cattle. The enriched GO terms and KEGG pathways revealed in response to maternal overnutrition underscore a complex cellular adaptation, particularly highlighting the significance of ‘extracellular matrix organization’, ‘cell surface receptor signaling pathway’, and the ‘PI3K-Akt signaling pathway’. These findings suggest a strategic cellular response aimed at stabilizing tissue architecture and signaling in response to metabolic stress.

The critical role of ECM in maintaining tissue integrity and facilitating cellular communication [46], combined with the PI3K-Akt pathway’s involvement in metabolic regulation [47], reflect a multifaceted approach to preserving cellular homeostasis. Recent studies add context to these findings: one showed that promoter hypomethylation related to maternal undernutrition was frequently observed in genes that participate in the PI3K-Akt signaling pathway and in some other ways, such as thyroid hormone signaling, Ras/Rap1 signaling, cAMP signaling, fatty acid metabolism, and cholesterol metabolism [28]. Moreover, another study identified skeletal muscle genes involved in the PI3K/Akt pathway as being significantly influenced by maternal protein supplementation during mid-gestation [13].

In addition to these pathways, our findings underscore the reprogramming of the liver’s transcriptional response to stress, as evidenced by the significant enrichment of the ‘transcription factor AP-1 complex’. The AP-1 complex plays a pivotal role in regulating gene expression in response to physiological and environmental stressors, mediating the liver’s adaptive responses to overnutrition. This transcription factor complex, which includes components such as Jun and Fos, orchestrates a wide array of cellular processes, including proliferation, differentiation, and apoptosis [48,49], and is crucial for the liver’s ability to manage and mitigate the metabolic challenges imposed by excessive maternal nutrition. The upregulation of *HSPA6*, within this context, underscores its potential role in fortifying cellular defenses against the biochemical alterations induced by overnutrition, pointing to its importance in mediating developmental and metabolic resilience in offspring exposed to maternal dietary excess.

SAT development in fetal beef cattle commences early in gestation, involving the transformation of mesenchymal stem cells into adipocytes and fibroblasts, a process influenced by nutrition, among other factors [37]. The role of maternal nutrition is crucial, as it directly impacts adipocyte differentiation, thereby affecting fetal development and the offspring’s metabolic health. Optimal maternal nutrition is vital for enhancing adipose tissue, which is of significant interest in the beef industry due to its direct correlation with meat quality traits such as marbling, tenderness, and flavor [37]. Our findings indicate that maternal overnutrition leads to the upregulation of genes like *WNT16*, *LOX*, *FBLN1*, *SLC2A3*, and *ABCG1*, among others, which are more closely associated with adipocyte development in SAT. *WNT16* is implicated in adipose differentiation in cattle [50]; *LOX*, a crucial enzyme for extracellular matrix integrity, promotes adipogenesis by inhibiting FGF-2 signaling pathways [51]. *FBLN1*, as part of the extracellular matrix [52], may influence adipocyte differentiation. *SLC2A3*, originally identified as a glucose transporter found predominantly in the nervous system [53], has functions extending beyond neuronal glucose transport to include essential roles in adipocyte energy supply and lipid synthesis [54]. Furthermore, the elevated expression of *ABCG1* observed in our study may play a crucial role in lipid transport and cholesterol homeostasis in the SAT of neonatal calves, as similar upregulation has been observed in dairy cows during late lactation to manage cholesterol levels effectively [55]. The enriched terms, such as ‘extracellular matrix organization’ and ‘cell differentiation’, alongside pathways like the ‘PI3K-Akt signaling pathway’ and ‘ECM–receptor interaction’, underscore the complex cellular orchestration at play. These components highlight the intricate environment that supports adipocyte maturation and SAT formation, suggesting that maternal overnutrition not only modulates isolated genes, but also a network of pathways crucial for adipose tissue development. Specifically, the upregulation of components involved in the extracellular matrix and signaling pathways provides a scaffold and the signals essential for adipocyte differentiation and function.

The findings showed that several genes connected to fibrogenesis and the differentiation of mesenchymal stem cells were upregulated in the SAT, including collagen genes (*COL1A1*, *COL1A2*, *COL3A1*, *COL4A5*, *COL4A6*, *ADAMTS2*), integrins (*ITGBL1*, *ITGA11*), and some others, such as *TGFBR3*, *FN1,* and *MMP14*. Collagen genes play a pivotal role in fibrogenesis within adipose tissue, providing structural integrity, regulating adipocyte differentiation, and facilitating extracellular matrix remodeling, which influence metabolic functionality [56]. For instance, a study on Nellore cows showed that the control group was fed at maintenance levels (100%) and the overfed group was fed at 1.5 times maintenance (150%); the results indicated that maternal overnutrition significantly increased *COL3A1* expression, indicative of enhanced fibrogenesis, while *COL1A1* expression remained unaffected across different gestational periods in fetal muscle tissues [57]. Integrins are crucial in fibrogenesis, mediating interactions between cells and the ECM [58]. The upregulated GO terms and KEGG pathways like “ECM–receptor interaction,” “Focal adhesion,” and “PI3K-Akt signaling pathway” indicate a complex interaction between the cellular components and signaling pathways essential for fibrogenesis and tissue remodeling. In our study, the PPI network analysis using significant DEGs from SAT not only highlighted the well-known structural components like collagen genes such as *COL1A1* and *COL1A2*, alongside integrins like *ITGBL1* and *ITGA11*, but also brought to light the critical roles of the enzyme *ADAMTS2*. *ADAMTS2* is essential for the maturation of collagen fibers, which is crucial for the structural integrity and functionality of the extracellular matrix [59]. This enzymatic action is particularly significant in the context of fibrogenesis and adipocyte differentiation, which are influenced by maternal overnutrition. Interestingly, the chemokine *CCL2* was identified as downregulated in the network, which is involved in the attraction of monocytes to sites of inflammation [60]. This reduction in *CCL2* expression may reflect a regulatory adaptation in adipose tissue in response to maternal overnutrition, potentially moderating inflammatory responses within adipose tissues. Such a decrease could be protective, aiming to mitigate excessive inflammation which might otherwise adversely affect tissue functionality and differentiation processes. This analysis, coupled with the observed regulation of specific GO terms and KEGG pathways related to extracellular matrix interactions and signaling, underscores the multifaceted regulatory mechanisms governing fibrogenesis in the SAT. These insights offer a deeper understanding of the biological intricacies of adipose tissue development and their implications for meat quality in beef cattle.

## 5. Conclusions

Our study on the effects of maternal overnutrition on the transcriptomic responses in the SAT and liver of newborn Hanwoo calves has demonstrated significant molecular adaptations. Notably, there was substantial upregulation of genes related to stress response mechanisms, such as *HSPA6* and *HSPA1A*, indicating a robust cellular mechanism to combat the physiological challenges posed by increased maternal nutrition. This upregulation underscores the activation of stress response pathways, potentially serving as a universal mechanism to cope with metabolic stress. Additionally, genes involved in adipogenesis and fibrogenesis were also significantly affected in the SAT. The integration of GO terms, KEGG pathways, and PPI networks further elaborates on the complex cellular adaptations occurring in response to maternal overnutrition. Key pathways such as the ‘extracellular matrix organization,’ ‘PI3K–Akt signaling pathway’, and ‘cell surface receptor signaling pathway’ were particularly enriched, not only contributing to immediate tissue responses but also potentially dictating long-term developmental patterns in offspring, affecting their growth, health, and productivity. Despite these insightful findings, the study’s limited sample size, particularly for the SAT analysis, may constrain the generalizability of the results. Future research should focus on alternative approaches, such as longitudinal studies or advanced in vitro models, to confirm and expand these transcriptomic insights.

## Figures and Tables

**Table 1:** Composition and nutritional content of diets used for Hanwoo cows in the study.

Item	Concentrate	Rice Straw
Dry Matter (%)	88.43	97.87
	% of Dry Matter	
Crude Protein	16.43	6.22
TDN ^1^	75.40	60.64
NDF ^2^	35.64	71.47
ADF ^3^	13.83	38.56
Ash	7.55	11.31
Ether extract	3.42	0.64
Crude fiber	8.14	29.99
Lignin	2.88	4.50

^1^ Total digestible nutrients; ^2^ neutral detergent fiber; ^3^ acid detergent fiber.

## Data Availability

The data supporting the findings of this study can be obtained from the corresponding authors upon reasonable request.

## Supplementary Materials

The following supporting information can be downloaded at: https://www.mdpi.com/article/10.3390/genes15060704/s1, Table S1: Details of gene analysis in the SAT and liver tissues. Table S2: Enrichment of Gene Ontology (GO) terms and KEGG pathways in Differentially Expressed Genes (DEGs) from the SAT of newborn Hanwoo calves following maternal overnutrition. Table S3: Enrichment of Gene Ontology (GO) terms and KEGG pathways in Differentially Expressed Genes (DEGs) from the liver of newborn Hanwoo calves following maternal overnutrition.

## References

[1] King J.C. (2000). Physiology of pregnancy and nutrient metabolism. Am. J. Clin. Nutr..

[2] Sookoian S., Gianotti T.F., Burgueño A.L., Pirola C.J. (2013). Fetal metabolic programming and epigenetic modifications: A systems biology approach. Pediatr. Res..

[3] Diniz W.J., Crouse M.S., Cushman R.A., McLean K.J., Caton J.S., Dahlen C.R., Reynolds L.P., Ward A.K. (2021). Cerebrum, liver, and muscle regulatory networks uncover maternal nutrition effects in developmental programming of beef cattle during early pregnancy. Sci. Rep..

[4] Maloney C.A., Rees W.D. (2005). Gene-nutrient interactions during fetal development. Reproduction.

[5] Reynolds L.P., Ward A.K., Caton J.S. (2017). Epigenetics and developmental programming in ruminants: Long-term impacts on growth and development. Biology of Domestic Animals.

[6] Caton J.S., Crouse M.S., McLean K.J., Dahlen C.R., Ward A.K., Cushman R.A., Grazul-Bilska A.T., Neville B.W., Borowicz P.P., Reynolds L.P. (2020). Maternal periconceptual nutrition, early pregnancy, and developmental outcomes in beef cattle. J. Anim. Sci..

[7] Thompson R.P., Nilsson E., Skinner M.K. (2020). Environmental epigenetics and epigenetic inheritance in domestic farm animals. Anim. Reprod. Sci..

[8] Zhao L., Liu X., Gomez N.A., Gao Y., Son J.S., Chae S.A., Zhu M.-J., Du M. (2023). Stage-specific nutritional management and developmental programming to optimize meat production. J. Anim. Sci. Biotechnol..

[9] Reynolds L.P., Borowicz P.P., Caton J.S., Crouse M.S., Dahlen C.R., Ward A.K. (2019). Developmental programming of fetal growth and development. Vet. Clin. N. Am. Food Anim. Pract..

[10] Copping K., Hernandez-Medrano J., Hoare A., Hummitzsch K., McMillen I., Morrison J., Rodgers R., Perry V. (2020). Maternal periconceptional and first trimester protein restriction in beef heifers: Effects on placental parameters and fetal and neonatal calf development. Reprod. Fertil. Dev..

[11] Palmer E.A., Peñagaricano F., Vedovatto M., Oliveira R.A., Field S.L., Laporta J., Moriel P. (2021). Effects of maternal gestational diet, with or without methionine, on muscle transcriptome of *Bos indicus*-influenced beef calves following a vaccine-induced immunological challenge. PLoS ONE.

[12] Shao T., McCann J.C., Shike D.W. (2023). Effects of late gestation supplements differing in fatty acid amount and profile to beef cows on cow performance, steer progeny growth performance through weaning, and relative mrna expression of genes associated with muscle and adipose tissue development. Animals.

[13] Carvalho E.B., Costa T.C., Sanglard L.P., Nascimento K.B., Meneses J.A., Galvão M.C., Serão N.V., Duarte M.S., Gionbelli M.P. (2022). Transcriptome profile in the skeletal muscle of cattle progeny as a function of maternal protein supplementation during mid-gestation. Livest. Sci..

[14] Bonnet M., Cassar-Malek I., Chilliard Y., Picard B. (2010). Ontogenesis of muscle and adipose tissues and their interactions in ruminants and other species. Animal.

[15] Funston R.N., Larson D.M., Vonnahme K. (2010). Effects of maternal nutrition on conceptus growth and offspring performance: Implications for beef cattle production. J. Anim. Sci..

[16] Underwood K., Tong J., Price P., Roberts A., Grings E., Hess B., Means W., Du M. (2010). Nutrition during mid to late gestation affects growth, adipose tissue deposition, and tenderness in cross-bred beef steers. Meat Sci..

[17] Zhang Y., Otomaru K., Oshima K., Goto Y., Oshima I., Muroya S., Sano M., Saneshima R., Nagao Y., Kinoshita A. (2021). Effects of low and high levels of maternal nutrition consumed for the entirety of gestation on the development of muscle, adipose tissue, bone, and the organs of Wagyu cattle fetuses. Anim. Sci. J..

[18] Zhang Y., Otomaru K., Muroya S., Gotoh T. (2022). Maternal nutrition during gestation alters histochemical properties, and mRNA and microRNA expression in adipose tissue of wagyu fetuses. Front. Endocrinol..

[19] Jennings T., Underwood K., Wertz-Lutz A., Weaver A. (2010). Effect of Maternal Nutrition on Fetal Adipocyte Development. https://openprairie.sdstate.edu/sd_beefreport_2010/12/.

[20] Long N., Prado-Cooper M., Krehbiel C., DeSilva U., Wettemann R. (2010). Effects of nutrient restriction of bovine dams during early gestation on postnatal growth, carcass and organ characteristics, and gene expression in adipose tissue and muscle. J. Anim. Sci..

[21] Muhlhausler B.S., Duffield J., McMillen I. (2007). Increased maternal nutrition increases leptin expression in perirenal and subcutaneous adipose tissue in the postnatal lamb. Endocrinology.

[22] Hyatt M.A., Budge H., Symonds M.E. (2008). Early developmental influences on hepatic organogenesis. Organogenesis.

[23] Smith B.I., Liefeld A., Vásquez-Hidalgo M.A., Vonnahme K.A., Grazul-Bilska A.T., Swanson K.C., Mishra N., Reed S.A., Zinn S.A., Govoni K.E. (2021). Mid-to late-gestational maternal nutrient restriction followed by realimentation alters development and lipid composition of liver and skeletal muscles in ovine fetuses. J. Anim. Sci..

[24] Hyatt M., Gardner D., Sebert S., Wilson V., Davidson N., Nigmatullina Y., Chan L., Budge H., Symonds M. (2011). Suboptimal maternal nutrition, during early fetal liver development, promotes lipid accumulation in the liver of obese offspring. Reproduction.

[25] Hyatt M., Gopalakrishnan G., Bispham J., Gentili S., McMillen I., Rhind S., Rae M., Kyle C., Brooks A., Jones C. (2007). Maternal nutrient restriction in early pregnancy programs hepatic mRNA expression of growth-related genes and liver size in adult male sheep. J. Endocrinol..

[26] Prezotto L., Camacho L., Lemley C., Keomanivong F., Caton J., Vonnahme K., Swanson K. (2016). Nutrient restriction and realimentation in beef cows during early and mid-gestation and maternal and fetal hepatic and small intestinal in vitro oxygen consumption. Animal.

[27] Diniz W.J., Ward A.K., McCarthy K.L., Kassetas C.J., Baumgaertner F., Reynolds L.P., Borowicz P.P., Sedivec K.K., Kirsch J.D., Dorsam S.T. (2023). Periconceptual maternal nutrition affects fetal liver programming of energy- and lipid-related genes. Animals.

[28] Muroya S., Otomaru K., Oshima K., Oshima I., Ojima K., Gotoh T. (2023). DNA Methylation of Genes Participating in Hepatic Metabolisms and Function in Fetal Calf Liver Is Altered by Maternal Undernutrition during Gestation. Int. J. Mol. Sci..

[29] NIAS (2017). Korean Feeding Standard for Hanwoo.

[30] Park M.S., Shokrollahi B., Kim U.H., Won J.I., Cho S.-H., Jin S., Kang S.S., Moon S.J., Um K.-H., Jang K.S. (2023). Effects of mid-to-late prepartum feed supplementation in Hanwoo beef cows on their performance, blood metabolites, and the carcass characteristics and metabolites of their neonatal calves. Front. Vet. Sci..

[31] Underwood W., Anthony R. (2020). AVMA Guidelines for the Euthanasia of Animals: 2020 Edition.

[32] Andrews S. (2010). FastQC: A Quality Control Tool for High Throughput Sequence Data.

[33] Kim D., Langmead B., Salzberg S.L. (2015). HISAT: A fast spliced aligner with low memory requirements. Nat. Methods.

[34] Love M.I., Huber W., Anders S. (2014). Moderated estimation of fold change and dispersion for RNA-seq data with DESeq2. Genome Biol..

[35] Raudvere U., Kolberg L., Kuzmin I., Arak T., Adler P., Peterson H., Vilo J. (2019). g:Profiler: A web server for functional enrichment analysis and conversions of gene lists (2019 update). Nucleic Acids Res..

[36] Von Mering C., Jensen L.J., Snel B., Hooper S.D., Krupp M., Foglierini M., Jouffre N., Huynen M.A., Bork P. (2005). STRING: Known and predicted protein–protein associations, integrated and transferred across organisms. Nucleic Acids Res..

[37] Du M. (2023). Prenatal development of muscle and adipose and connective tissues and its impact on meat quality. Meat Muscle Biol..

[38] Herring C.M., Bazer F.W., Johnson G.A., Wu G. (2018). Impacts of maternal dietary protein intake on fetal survival, growth, and development. Exp. Biol. Med..

[39] Moura C.S., Lollo P.C.B., Morato P.N., Amaya-Farfan J. (2018). Dietary nutrients and bioactive substances modulate heat shock protein (HSP) expression: A review. Nutrients.

[40] Kuppuswami J., Senthilkumar G.P. (2023). Nutri-stress, mitochondrial dysfunction, and insulin resistance—Role of heat shock proteins. Cell Stress Chaperones.

[41] Hyder I., Pasumarti M., Reddy P.R., Prasad C.S., Kumar K.A., Sejian V. (2017). Thermotolerance in domestic ruminants: A HSP70 perspective. Heat Shock Proteins in Veterinary Medicine and Sciences.

[42] Banerjee D., Upadhyay R.C., Chaudhary U.B., Kumar R., Singh S., Ashutosh, Polley S., Mukherjee A., Das T.K., De S. (2014). Seasonal variation in expression pattern of genes under HSP70: Seasonal variation in expression pattern of genes under HSP70 family in heat-and cold-adapted goats (Capra hircus). Cell Stress Chaperones.

[43] Mohanarao G.J., Mukherjee A., Banerjee D., Gohain M., Dass G., Brahma B., Datta T.K., Upadhyay R.C., De S. (2014). HSP70 family genes and HSP27 expression in response to heat and cold stress in vitro in peripheral blood mononuclear cells of goat (*Capra hircus*). Small Rumin. Res..

[44] Daugaard M., Rohde M., Jäättelä M. (2007). The heat shock protein 70 family: Highly homologous proteins with overlapping and distinct functions. FEBS Lett..

[45] Rocha R.d.F.B., Baena M.M., de Cássia Estopa A., Gervásio I.C., Ibelli A.M.G., Gionbelli T.R.S., Gionbelli M.P., de Freitas R.T.F., Meirelles S.L.C. (2019). Differential expression of HSF1 and HSPA6 genes and physiological responses in Angus and Simmental cattle breeds. J. Therm. Biol..

[46] Frantz C., Stewart K.M., Weaver V.M. (2010). The extracellular matrix at a glance. J. Cell Sci..

[47] Li L., He M.L., Wang K., Zhang Y.S. (2018). Buffering agent via insulin-mediated activation of PI3K/AKT signaling pathway to regulate lipid metabolism in lactating goats. Physiol. Res..

[48] Marinho H.S., Real C., Cyrne L., Soares H., Antunes F. (2014). Hydrogen peroxide sensing, signaling and regulation of transcription factors. Redox Biol..

[49] Schulien I., Hockenjos B., Schmitt-Graeff A., Perdekamp M.G., Follo M., Thimme R., Hasselblatt P. (2019). The transcription factor c-Jun/AP-1 promotes liver fibrosis during non-alcoholic steatohepatitis by regulating Osteopontin expression. Cell Death Differ..

[50] Pan C., Wang S., Yang C., Hu C., Sheng H., Xue X., Hu H., Lei Z., Yang M., Ma Y. (2022). Genome-wide identification and expression profiling analysis of Wnt family genes affecting adipocyte differentiation in cattle. Sci. Rep..

[51] Griner J.D., Rogers C.J., Zhu M.-J., Du M. (2017). Lysyl oxidase propeptide promotes adipogenesis through inhibition of FGF-2 signaling. Adipocyte.

[52] Olijnyk D., Ibrahim A., Ferrier R., Tsuda T., Chu M.-L., Gusterson B., Stein T., Morris J. (2014). Fibulin-2 is involved in early extracellular matrix development of the outgrowing mouse mammary epithelium. Cell. Mol. Life Sci..

[53] Ziegler G.C., Almos P., McNeill R.V., Jansch C., Lesch K.P. (2020). Cellular effects and clinical implications of SLC2A3 copy number variation. J. Cell. Physiol..

[54] Dos Santos Silva D.B., Fonseca L.F.S., Pinheiro D.G., Muniz M.M.M., Magalhães A.F.B., Baldi F., Ferro J.A., Chardulo L.A.L., De Albuquerque L.G. (2019). Prediction of hub genes associated with intramuscular fat content in Nelore cattle. BMC Genom..

[55] Kessler E.C., Gross J.J., Bruckmaier R., Albrecht C. (2014). Cholesterol metabolism, transport, and hepatic regulation in dairy cows during transition and early lactation. J. Dairy Sci..

[56] Ruiz-Ojeda F.J., Méndez-Gutiérrez A., Aguilera C.M., Plaza-Díaz J. (2019). Extracellular matrix remodeling of adipose tissue in obesity and metabolic diseases. Int. J. Mol. Sci..

[57] Duarte M., Gionbelli M., Paulino P., Serão N., Nascimento C., Botelho M., Martins T., Filho S., Dodson M., Guimarães S. (2014). Maternal overnutrition enhances mRNA expression of adipogenic markers and collagen deposition in skeletal muscle of beef cattle fetuses. J. Anim. Sci..

[58] Hayashida T. (2010). Integrins modulate cellular fibrogenesis at multiple levels: Regulation of TGF-β signaling. Endocr. Metab. Immune Disord. Drug Targets.

[59] Liao H., Zhang X., Qi Y., Wang Y., Pang Y., Zhang Z., Liu P. (2018). The relationships of collagen and *ADAMTS2* expression levels with meat quality traits in cattle. Indian J. Anim. Res..

[60] Gschwandtner M., Derler R., Midwood K.S. (2019). More than just attractive: How CCL2 influences myeloid cell behavior beyond chemotaxis. Front. Immunol..

